# Prospective analysis of bleomycin electrosclerotherapy for clinical outcome and volume reduction in therapy refractory slow-flow malformations

**DOI:** 10.1186/s42155-025-00641-z

**Published:** 2025-12-31

**Authors:** Anna Deleu, Richard Brill, Marie-Sophie Schüngel, Julius H. Loeser, Oleksandr Bidakov, Moritz Guntau, Vanessa F. Schmidt, Moritz Wildgruber, Constantin Goldann, Walter A. Wohlgemuth

**Affiliations:** 1 University Clinic and Polyclinic of Radiology, Universitätsklinikum Halle, Halle (Saale), Germany; 2 TUM School of Medicine and Health, Technical University Munich, Munich, Germany; 3 Department of Diagnostic and Interventional Radiology, Faculty of Medicine, University Hospital Wuerzburg, University of Wuerzburg, Würzburg, Germany; 4 Department of Radiology, LMU University Hospital, LMU Munich, Munich, Germany; 5 Institut für Diagnostische und Interventionelle Radiologie und Neuroradiologie, München Klinik Harlaching, Akademisches Lehrkrankenhaus Ludwig-Maximilians Universität München, Munich, Germany

**Keywords:** Bleomycin, Electrosclerotherapy, Vascular malformations, Venous malformations, Lymphatic malformations

## Abstract

**Background:**

Slow-flow vascular malformations are persistent congenital vascular lesions that progressively disrupt tissue structure and function, often causing pain, swelling, and esthetic concerns. Despite the availability of surgical and sclerotherapy-based interventions, treatment outcomes are often unsatisfactory, with high rates of recurrence and resistance. The aim of this study was to prospectively evaluate the safety and effectiveness of bleomycin electrosclerotherapy in reducing lesion volume, alleviating symptoms, and improving clinical outcomes in 33 patients with slow-flow vascular malformations resistant to previous treatments. The prospective design allowed real-time observation of patients’ responses to therapy, while the longitudinal follow-up, beginning with recruitment in 2020 and continuing through the end of 2024, allowed comprehensive monitoring of outcomes.

**Results:**

After one treatment session, 33 of 35 (94.3%) lesions demonstrated symptomatic improvement, and all lesions (*n* = 35/35, 100%) showed a reduction in volume. Average volume decreased from 1781.1 to 1335.0 mL (25.0%) after one session and 1189.13 mL (33.24%) after final treatment. Mild adverse events, including redness (*n* = 4) and swelling (*n* = 25), resolved within 4 weeks. Skin changes like hyperpigmentation (*n* = 3) and livid discoloration (*n* = 4) could be observed for longer periods of time.

**Conclusions:**

Bleomycin electrosclerotherapy demonstrated high effectiveness and safety for treating slow-flow malformations, establishing it as a promising therapeutic option even for lesions that have responded insufficiently to previous treatment attempts.

**Supplementary Information:**

The online version contains supplementary material available at 10.1186/s42155-025-00641-z.

## Background

Slow-flow malformations are congenital venous anomalies that can occur in the skin, subcutaneous tissue, muscles, or around internal organs, often causing pain and discomfort with symptoms varying by location [1]. According to the International Society for the Study of Vascular Anomalies (ISSVA) [2], slow-flow vascular malformations include venous, lymphatic, and mixed malformations. Sclerotherapy is the most widely used treatment for slow-flow malformations [3]. Common sclerosing agents in clinical use are pingyangmycin, absolute ethanol, OK-432, ethanolamine oleate, and bleomycin. These agents generally demonstrate good efficacy, with complete response rates of 39–76% and overall response rates of 71–98%. However, some agents, especially absolute ethanol, are associated with severe complications such as skin necrosis and nerve injury, limiting their clinical use. Bleomycin and ethanolamine oleate offer similar efficacy with a lower risk of serious adverse events (AE) [4]. Bleomycin is a well-established sclerosing agent though its effectiveness is limited by the size and charge of its molecule, which restricts cellular permeability, and a risk for lung toxicity when used in high doses. In addition, bleomycin sclerotherapy without electroporation generally leads to only modest clinical improvement. Patient satisfaction tends to be moderate to low, and most patients report interest in undergoing additional treatment. Moreover, complete response rates are limited and show substantial variability, ranging from 20 to 57% [4–7]. Electroporation is a technique that applies electric pulses to create temporary pores in the cell membrane, allowing for the introduction of DNA, chemicals, or drugs into cells. The outcome of electroporation depends on the intensity and number of pulses, with higher strengths leading to greater permeability [8]. Combining bleomycin with electroporation offers a new strategy to enhance its efficacy in the treatment of slow-flow vascular malformations. In bleomycin electrosclerotherapy (BEST), electroporation facilitates the uptake of bleomycin by increased cell membrane permeability, leading to higher intracellular drug concentrations compared to conventional sclerotherapy with bleomycin [9]. Electroporation, in combination with intravenous or directly injected cytotoxic drugs, has proven to be a successful treatment option in interventional oncology for various malignancies [10–12]. The extension of the method beyond oncology into the realm of vascular malformations represents an evolution in its application. In this prospective study, which adds to the limited existing data and dedicated imaging analyses in the literature, we analyzed the effectiveness of BEST, in terms of volume reduction and clinical outcome, to treat slow-flow malformations in patients who had been resistant to previous therapy.

## Methods

### Study design and patient characteristics

**Fig. 1:**
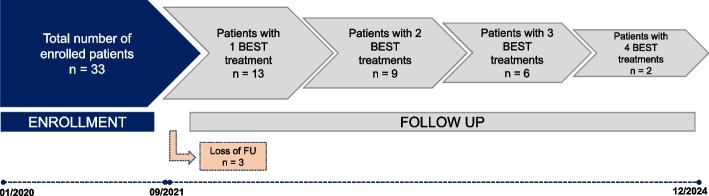
Flow chart of the study design

From 2020 to 2021, 33 patients with symptomatic slow-flow vascular malformations were enrolled at the University Clinic and Polyclinic of Radiology, Universitätsklinikum Halle (Saale), Germany. All participants were recruited and provided informed consent prior to the initiation of therapy, and data were collected throughout the follow-up period. Eligible patients (*n* = 30) had a confirmed slow-flow vascular malformation, underwent at least one BEST session, and completed at least one follow-up. Patients unable to attend follow-ups or undergo MRI were excluded (*n* = 3) (Fig. 1). All included patients were therapy refractory, having previously received treatments that did not improve symptoms or reduce lesion volume. Most patients (*n* = 25, 83.3%) had one lesion to get treated, while 5 (16.6%) patients had two lesions treated in separate sessions, totaling 35 lesions (27 venous, 8 veno-lymphatic). The mean lesion volume at baseline was 1781.1 mL, ranging from 1.5 to 15,931.2 mL. The number of sessions was guided by therapeutic response and residual lesion size, with follow-up continuing through the end of 2024. More details are given in Supplementary File 1.

### Bleomycin electrosclerotherapy

**Fig. 2:**
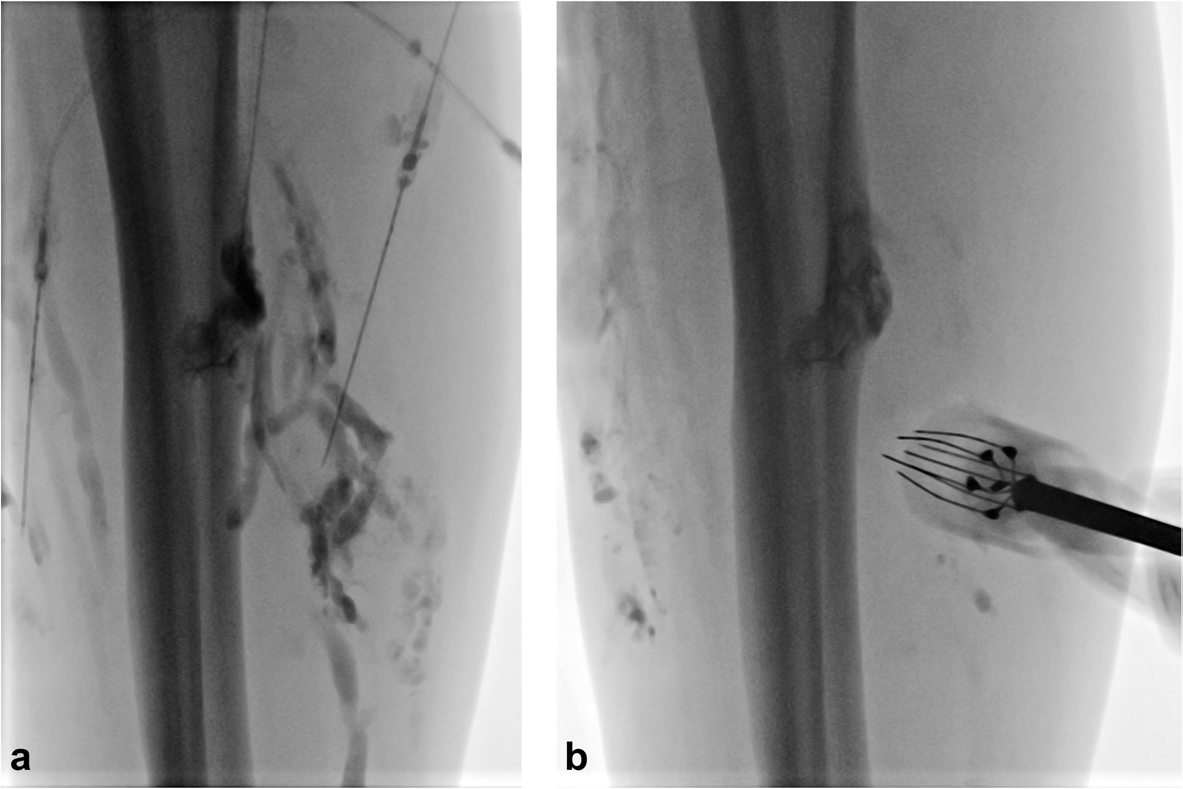
Digital subtraction angiography during initial BEST session. Digital subtraction angiography (DSA) acquired during the initial BEST session of a patient with a venous malformation on the right lower leg. **a** Contrast-enhanced filling of the dysplastic venous structures within the lesion. **b** Position of the hexagonal electrode during treatment

BEST was administered following the latest clinical operating procedures [13]: for extensive venous malformations, preoperative evaluation included D-dimer and fibrinogen testing. Low fibrinogen prompted low molecular weight heparin (LMWH) 5 days before treatment; otherwise, LMWH was started on the procedure day and continued for 7 days. This approach was adopted to reduce the risk of peri-interventional thromboembolic events. The procedure involved intralesional bleomycin injection, electrode placement, and delivery of short electric pulses for reversible electroporation. After direct percutaneous intralesional injection of a contrast agent into the slow-flow malformation under fluoroscopic guidance, a 0.25 mg/mL (250 IU/mL) solution of bleomycin and contrast agent (1:3 dilution) was injected. The volume of bleomycin injected was adapted to the lesion size (determined by its longest diameter) and drainage pattern, as larger or more complex malformations with extensive drainage required greater volumes to achieve adequate intralesional distribution. Care was taken to ensure that the total dose per session did not exceed 10,000 IU. Electrodes were selected and inserted based on lesion depth and size before pulses were delivered using the Cliniporator VITAE system (Fig. 2). Interventions were performed under general anesthesia due to the painful electric pulses. To minimize the risk of hyperpigmentation, tape use for electrocardiogram (ECG) stickers and other skin fixations was kept to a minimum, and removal was performed carefully. After bleomycin administration, FiO_2_ was maintained below 30% whenever possible. More details are given in Supplementary File 2.

### Follow-up and definition of clinical success

All patients underwent the same protocol, including a medical history interview and clinical examination, as well as ultrasound and MRI assessment at each follow-up visit. Documentation included the date and number of sessions, treated location, type of electrodes used, injected and cumulative dose of bleomycin, and any encountered technical problem. Clinical response was defined as a change in symptoms (pain, swelling, impaired physical function, reduced sensitivity, wounds, skin discoloration) documented at the post-therapy follow-up outpatient visit. Response assessment was based on pain scales, ultrasound, and clinical examination. The clinical response was categorized as asymptomatic, improved, unchanged, or worsened. Clinical success was defined as patient being asymptomatic or reporting improved symptoms at follow-up.

### Definition of adverse events

All post-therapy symptoms, documented as AE and occurring during the intervention and clinical follow-up, were analyzed according to the classification system of the Cardiovascular and Interventional Radiological Society of Europe (CIRSE) [14]. Special attention was paid to anticipated symptoms such as prolonged swelling, skin redness, and both local and generalized persistent pain. No patient experienced unexpected AE.

### MRI volumetric analysis

All patients underwent MRI on a 3-Tesla whole-body MR scanner (Magnetom Skyra, Siemens Healthineers, Erlangen, Germany) using a standardized vascular malformation protocol (provided in Supplementary File 3). The lesions were measured manually at baseline before the first BEST intervention and again at the post-therapy follow-up visits. Given the irregular morphology of slow-flow vascular malformations, the lesion volume was approximated using a rotational ellipsoid formula ($V=\frac{\pi }{6}\times {d}_{e}^{2}\times {d}_{p}$), in which ${d}_{e}$ represents the equatorial diameter as well as ${d}_{p}$ represents the polar diameter. The relative change in volume between the baseline scans obtained before the intervention and follow-up scans was calculated.

## Results

### Patient characteristics

A total of 30 patients (17 males and 13 females, median age: 30 years, range: 18–61 years) with extracranial, slow-flow vascular malformations were included in this study. All lesions were previously treated with multiple therapies including surgery (*n* = 13/35, 37.1%), sclerotherapy without bleomycin (*n* = 34/35, 97.1%), laser therapy (*n* = 7/35, 20%), or compression therapy (*n* = 7/35, 20%) without achieving adequate symptom relief. Symptoms linked to the presence of the lesions included swelling (*n* = 31/35, 88.6%), skin changes (such as redness and livid discolorations; *n* = 27/35, 77.1%), physical (*n* = 17/35, 48.6%) and sensitivity impairment (*n* = 2/35, 5.7%), and hypotrophy (*n* = 4/35, 11.4%). Patients were monitored at follow-up at a mean of 8.65 months (range 3–32 months, SD 6.4 months). Table 1 provides a summary of the patients’ characteristics, while Table 2 details their pre-treatment symptoms including a detailed overview of the skin changes.

**Table 1:** Patients’ clinical characteristics. The table provides an overview of the clinical characteristics of 30 patients, with a median age of 30 years at first treatment. Most patients were male and presented with venous malformations, most commonly located in the lower extremities and head/neck regions. Most patients could benefit from 2 to 4 therapy sessions. Prior treatments included sclerotherapy in many cases, as well as surgery in several others. Abbreviations: *VMs*, venous malformations; *LMs*, lymphatic malformations; *CVLMs*, combined venous-lymphatic malformations; *CVS*, combined venous malformations; *VLM*, venous-lymphatic malformations

Parameters	Cohort (*n* = 30)
Age at first treatment, median (range)	30 (18–61)
Gender	
Male	17 (56.6%)
Female	13 (43.3%)
Malformations (*n* = 35)	
VMs	27 (77.1%)
LMs	0
CVLMs	2 (5.71%)
CVM	3 (8.57%)
VLM	3 (8.57%)
Anatomical site (*n* = 35)	
Head/neck	9 (25.71%)
Lower extremity	19 (54.28%)
Upper extremity	2 (5.71%)
Trunk/buttock	5 (14.28%)
Number of interventions	
1	14 lesions/13 patients
2	10 lesions/9 patients
3	9 lesions/6 patients
4	2 lesions/2 patients
Previous therapies	
Surgery	13
Non-BEST sclerotherapy	34
Laser	7
Compression	7

**Table 2:** Symptoms before and after BEST. The table summarizes patient-reported symptoms before and after BEST treatment. Swelling, skin changes, and functional limitations were initially the most frequent symptoms. These symptoms showed reduction after the first treatment session and continued to decrease by the final session, demonstrating overall improvements in swelling, skin condition, and function, and highlighting BEST’s effectiveness in symptom relief

Symptoms	Before BEST	After 1st BEST	After last BEST
Swelling	31	25	19
Skin changes			
Hyperpigmentation	0	5	3
Redness	6	4	0
Livid discoloration	16	6	4
Varicose veins	8	3	0
Superficial vascular markings	6	3	3
Petechiae	1	1	0
Dry skin	0	1	0
Motor limitations	17	14	12
Reduced sensitivity	2	0	0
Thrombosis	3	0	0
Hypotrophy	4	0	1

### Procedural details

All BEST procedures were performed under general anesthesia. The mean injection volume of bleomycin solution was 4.82 mL per treatment (range: 0.25–15 mL, standard deviation, 4.1 mL). Given a concentration of 0.25 mg/mL (equivalent to 250 IU/mL), the corresponding mean administered bleomycin dose was 1.20 mg per treatment (range: 0.06–3.75 mg; standard deviation, 1.02 mg). Hexagonal electrodes were most commonly used (54.84% of the cases) followed by finger electrodes (25.81% of the cases). The median insertion depth of the used electrodes was 20 mm (range 10–40 mm). A total of 14 lesions (40.0%) required only a single therapy session, resulting in considerable volume reduction and symptom relief. Additional sessions were performed if the patient continued to experience symptoms and requested further treatment, as discussed during follow-up visits, especially if further volume reduction was expected. This applied to 21 lesions (60.0%) that underwent between 2 and 4 therapy sessions. Of these, 10/21 lesions (47.6%) were larger (volume > 500 mL) malformations that benefited from multiple treatment sessions. In these cases, bleomycin was incrementally injected at multiple intralesional sites to achieve optimal therapeutic outcomes.

### Treatment results

#### Volumetry

**Fig. 3:**
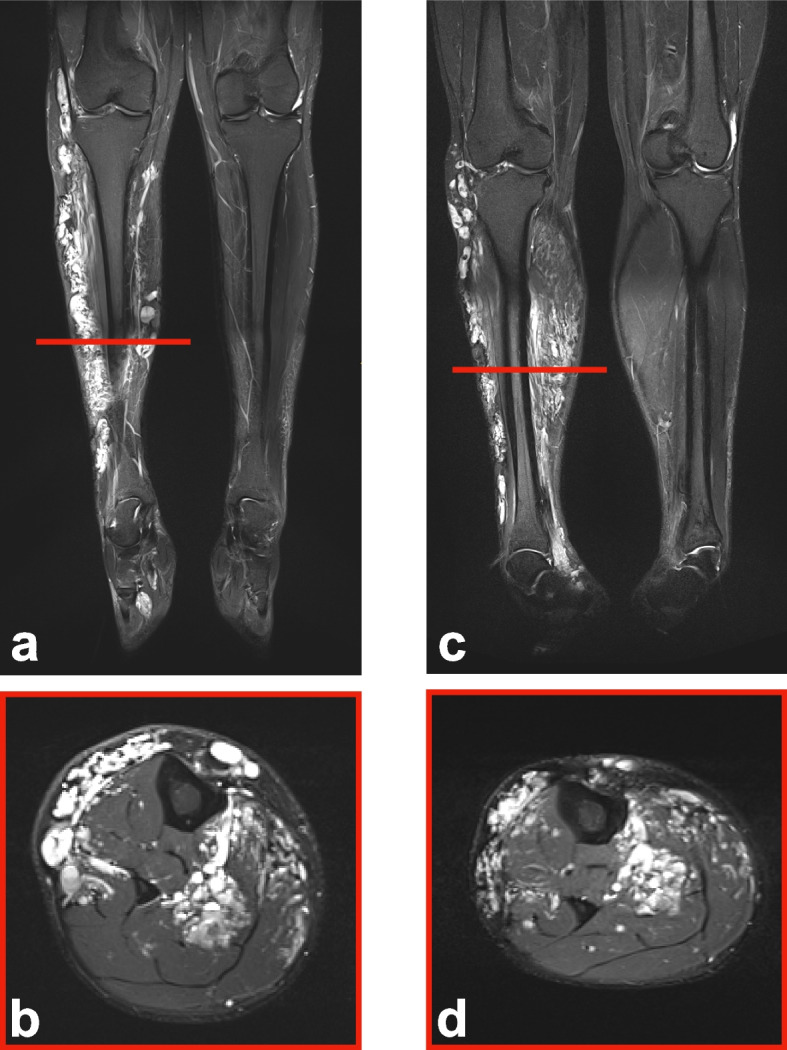
MRI imaging of venous malformation before and after BEST therapy. MRI depicting the case of a 27-year-old patient with a venous malformation on his right lower leg, treated with BEST. **a** Coronal MRI scan before treatment showing the lesion as a hyperintense area. **b** Axial MRI scan depicting the venous malformation at its maximum extent. **c** Coronal MRI scan of the leg obtained 4 months post-therapy, showing a reduction in lesion volume after just one therapy session, with notable decrease of the hyperintense area. **d** Axial MRI scan of the leg

**Fig. 4:**
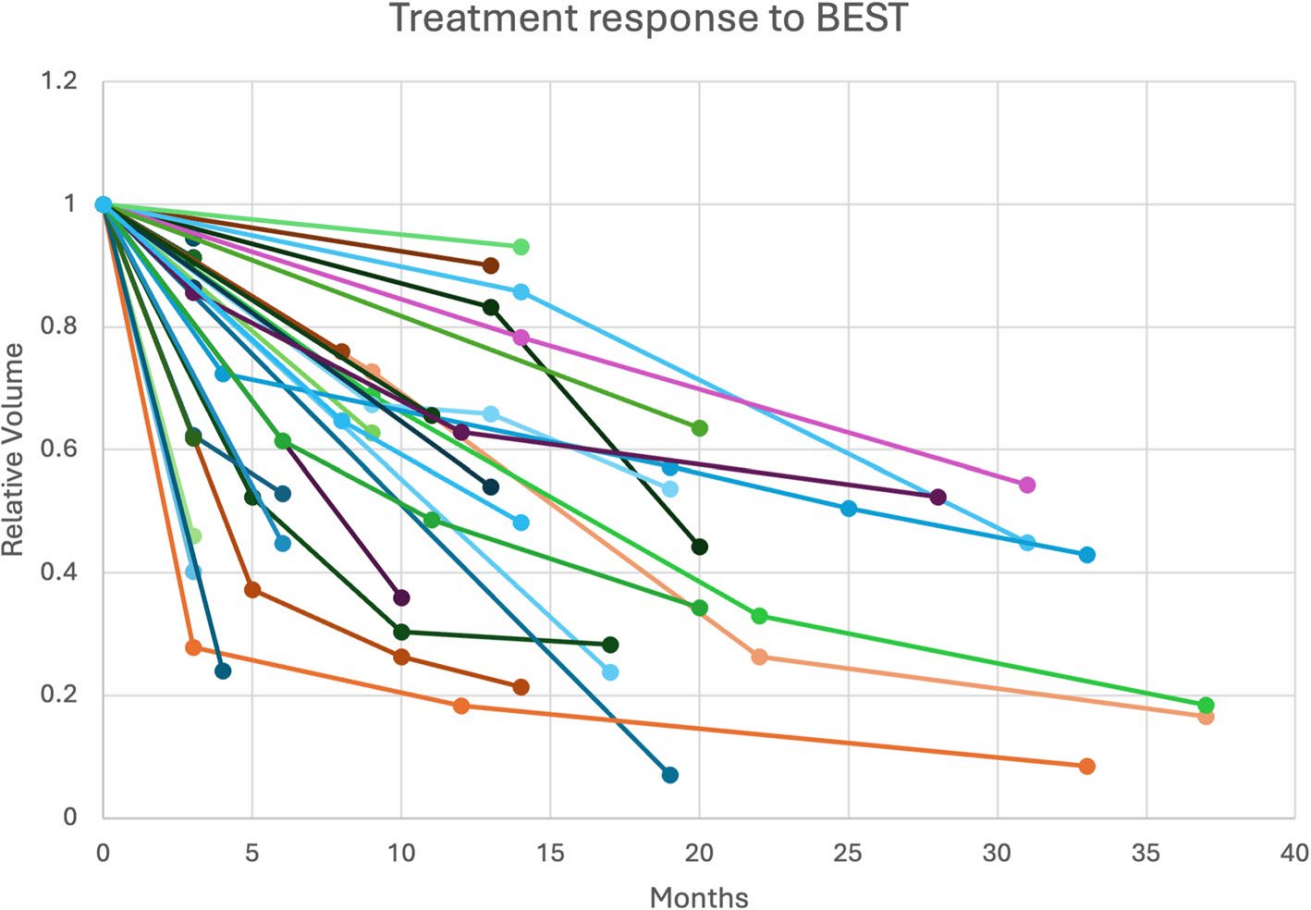
Treatment response to BEST. Graphical representation of the reduction in lesion volume over time following BEST treatment. Each line represents a patient’s response, with the x-axis indicating months post-treatment and the y-axis showing lesion volume relative to the volume prior to the first BEST session. While all patients experience volume reduction, the rate of reduction varies, highlighting the effectiveness of BEST across multiple sessions

**Fig. 5:**
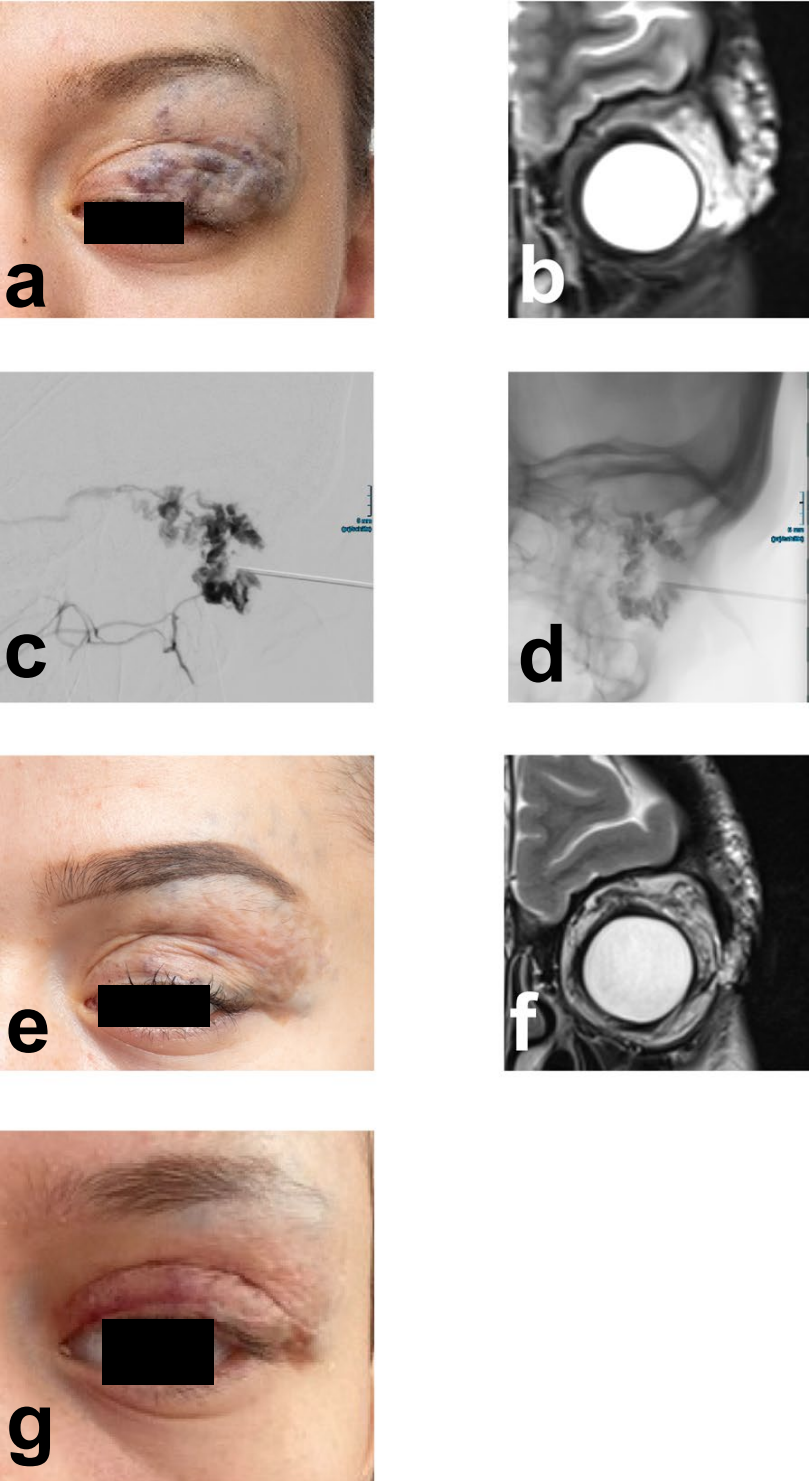
Progressive improvement of venous malformation on the eyelid under BEST therapy. A female patient (age 20 at her first session) underwent treatment with BEST for a vascular malformation of the eyelid. Although the lesion exhibited a relatively modest initial volume (33.5 mL), it resulted in functional impairment and had a marked cosmetic impact, both of which adversely affected the patient’s quality of life. Following three sessions of BEST, the lesion volume was reduced to 5.5 mL, representing an 83.6% reduction. This substantial volumetric reduction led to a substantial clinical improvement alleviating functional deficits and enabling subsequent surgical intervention for further esthetic refinement. **a** Patient’s condition prior to treatment, highlighting discoloration and swelling and **b** the corresponding MRI of the lesion. **c** and **d** Digital subtraction angiography showing the injection of bleomycin during BEST for the treatment of a lesion on the eyelid. **e** Final follow-up after 2 years and 4 months, demonstrating a notable reduction in lesion volume and swelling (**f**) as seen on MRI. The clinical photograph also highlights smoother skin texture and less discoloration. **g** Post-procedural image showing the patient after BEST, followed by eyelid correction surgery to restore symmetry

The mean lesion volume decreased from 1781.1 to 1335.0 mL (SD: 2732.8 mL) after the first session, representing a 25.0% volume reduction. After the final treatment, the mean volume was reduced to 1189.13 mL (SD: 2677.9), representing a 33.4% total reduction. At follow-up, clinical and imaging assessments suggested a general trend toward reduced malformation volume across patients, regardless of initial lesion size, anatomical location, or age (Figs. 3, 4, and 5).

#### Clinical outcome

The depth of application and the dose of injected bleomycin were adjusted for each session based on the specific circumstances and clinical evaluation of the patient. Prior to initiating BEST therapy, the most reported symptoms were swelling (*n* = 31), skin changes (*n* = 27), and restriction of motion (*n* = 17). Additional symptoms included livid discoloration (*n* = 16), varicose veins (*n* = 8), superficial vascular markings (*n* = 6), redness (*n* = 6), hypotrophy (*n* = 4), thrombosis (*n* = 3), sensitivity limitations (*n* = 2), and petechiae (*n* = 1) (Table 2). Importantly, no cases of thrombosis, sensitivity limitations, or hypotrophy were reported after the initial session. Following the first session of BEST, temporary symptoms were reported by several patients. Swelling was noted in 25/35 (71.4%) cases, skin changes in 21/35 (60.0%) cases, and restriction of motion in 14/35 (40.0%) cases. Other transient symptoms included livid discoloration (*n* = 6/35, 17.1%), hyperpigmentation (*n* = 5/35, 14.3%), redness (*n* = 4/35, 11.4%), varicose veins (*n* = 3/35, 8.6%), superficial vascular markings (*n* = 3/35, 8.6%), petechiae (*n* = 1/35, 2.9%), and dry skin (*n* = 1/35, 2.9%). These were managed symptomatically, when necessary, for example, with compression therapy in cases of swelling. Following the final BEST session, a further reduction in temporary symptoms was observed. Swelling persisted in 19/35 (54.3%) lesions, skin changes in 17/35 (48.6%) lesions, restriction of motion in 12/35 (34.3%) lesions, and livid discoloration in 4/35 (11.4%) lesions. Hyperpigmentation (*n* = 3/35, 8.6%), superficial vascular markings (*n* = 3/35, 8.6%), and hypotrophy (*n* = 1/35, 2.9%) were still reported, while redness, varicose veins, sensitivity limitations, and thrombosis were no longer observed (Table 2). Most lesions (*n* = 33/35, 88.57%) exhibited a reduction in symptoms and, among these, 2 (*n* = 2/33, 6.1%) improved so notably that the patients became completely asymptomatic. All patients who experienced symptom improvement also demonstrated a corresponding reduction in lesion volume. One patient showed a decrease in lesion size but reported no improvement in symptoms following therapy.

#### Adverse events

No adverse events were reported at any stage of the treatment.

## Discussion

The combination of bleomycin with electroporation represents a promising new strategy to enhance bleomycin’s therapeutic effectiveness in the treatment of slow-flow vascular malformations. However, to date, clinical data supporting this approach remains limited. This prospective study aimed to further substantiate the therapeutic effectiveness of BEST through the treatment and the follow-up of 30 patients presenting slow-flow vascular malformations with diverse sizes, locations, and depths. It is worth to mention that all patients received previous therapies, including surgery, laser therapy, or non-BEST sclerotherapy, without achieving satisfactory results. Baseline lesion volume varied widely, demonstrating that BEST is effective for both small and large malformations and represents the most effective current option for therapy refractory slow-flow malformations of any size. In accordance with other studies [15, 16], the 35 lesions treated with BEST showed a volume reduction already after a single session, with an average decrease of 25%, demonstrating the effectiveness of BEST. In addition, in 94.3% of the cases, the lesions exhibited symptom improvement. Based on patients’ reports, the results suggest a generally positive subjective response across several domains, including mobility, sensation, pain, and esthetic outcomes. BEST confirmed to be effective in some cases with just one or two treatments, emphasizing its advantage over traditional bleomycin sclerotherapy without electroporation, which typically requires multiple sessions to achieve similar results in treating slow-flow vascular malformations [17]. For larger lesions affecting extensive body areas, multiple BEST sessions were performed to gradually reduce lesion volume. This approach allowed patients to recover between sessions and enabled stepwise volume reduction while closely monitoring clinical outcomes. Follow-up assessments considered both changes in lesion size and patients’ symptoms, as well as their interest in further therapy. Notably, multiple sessions were also effective for smaller lesions as described in the case of the patients with the vascular malformation of the eyelid (Fig. 5). This suggests that a multi-session approach can be effective in both large and small malformations, reflecting a cumulative effect of BEST over successive treatments. The wide variety of available electrodes (such as freely positionable needle electrodes) allows access to challenging areas during therapy. This is especially important for lesions in areas of functional or esthetic relevance. In BEST, the volume of bleomycin used is lower than in traditional bleomycin sclerotherapy. Doses up to 15 mg bleomycin per session are used in traditional bleomycin sclerotherapy [18], whereas in our BEST study the mean dose was only 1.2 mg per session (range 0.06–3.75 mg), offering an important advantage by minimizing the risk of potential side effects, such as lung toxicity [19, 20]. In our cohort, the resulting safety profile was characterized by a low complication rate, likely due to the lower bleomycin dosage and the decreased need for multiple sessions. None of the patients experienced long-term AE that did not resolve spontaneously. Reported side effects were moderate and temporary, including redness, localized hyperpigmentation, livid discoloration, and swelling at the treatment site, all of which resolved spontaneously within 4 weeks without additional intervention. The treatment’s effectiveness was further validated by patient-reported outcomes, particularly highlighting improvements in mobility and esthetic satisfaction. Although two patients in the study reported no perceived improvement in the post-therapy condition, most of the cohort confirmed positive post-treatment outcomes. Our findings on safety and effectiveness align with a larger retrospective multicenter study investigating subjective outcomes reporting improvements regarding pain, mobility, and esthetics after BEST [15]. Additionally, another study corroborated our findings, indicating that BEST has only manageable side effects and provides enhanced therapeutic outcomes, confirming BEST as a promising alternative to traditional sclerotherapy [21]. This study demonstrates that BEST was highly effective for the patients included in the cohort. Its prospective design allowed for real-time observation of patient responses to the therapy. Moreover, the longitudinal nature of the follow-up enabled comprehensive monitoring of patients’ outcomes over an extended period (up to 32 months), and the provision of multiple therapy sessions for some patients provided valuable insights into the treatment’s efficacy across different scenarios. These strengths contribute to the overall reliability of the study’s findings and underscore the potential of BEST as a viable treatment option for slow-flow vascular malformations, including therapy resistant ones. However, the study has few limitations. The small cohort, reflecting the rarity of slow-flow vascular malformations, single-center recruitment, and the highly specialized nature of our treatment center, limits generalizability of our findings and could introduce selection bias. Future prospective multicenter studies with larger populations could further expand and validate these observations. Heterogeneous regimens for large or widespread lesions, including variations in treatment intervals and prior therapies, hinder outcome comparison, highlighting the need for standardized protocols on session number, electrode length, and application series. In addition, follow-up intervals were inconsistent, partly due to COVID-19 disruptions and related loss to follow-up, and with only 4 years of follow-up, long-term durability and recurrence could not be assessed. Moreover, the absence of a control group highlights the need for future comparative research which would allow a clearer comparison of BEST with other therapeutic options. Furthermore, the ellipsoid formula used for volume calculation may have over- or underestimated irregularly shaped lesions, which could affect the accuracy. Finally, all procedures in this study required general anesthesia due to electric pulses, unlike conventional sclerotherapy, which is often performed under local anesthesia. This adds risks, complexity, and costs, limiting access for patients needing multiple treatments. It also requires specialized equipment, trained staff, and hospital facilities, restricting use to high-resource centers and reducing scalability compared to outpatient sclerotherapy.

## Conclusions

The combination of bleomycin sclerotherapy and reversible electroporation is technically feasible and has shown considerable benefits for patients with therapy refractory slow-flow vascular malformations. This approach led to a reduction in lesion size and alleviated symptoms without causing long-term side effects. These encouraging results highlight BEST as a promising therapeutic option that warrants further investigation in larger, controlled, and multicenter studies.

## Supplementary Information

Supplementary Material 1.

Supplementary Material 2.

Supplementary Material 3.

## Data Availability

Data will be made available on reasonable request.

## Abbreviations

AE: Adverse event
BEST: Bleomycin electrosclerotherapy
LMWH: Low molecular weight heparin
ECG: Electrocardiogram

## References

[1] Dasgupta R, Patel M. Venous malformations. Semin Pediatr Surg. 2014;23:198–202. 25241098 10.1053/j.sempedsurg.2014.06.019

[2] ISSVA classification for vascular anomalies. International Society for the Study of Vascular Anomalies, 2025. https://www.issva.org/classification.

[3] Schmidt VF, Masthoff M, Goldann C, et al. Percutaneous sclerotherapy of venous malformations of the hand: a multicenter analysis. Cardiovasc Intervent Radiol. 2021;44:1543–50. 34286368 10.1007/s00270-021-02926-x PMC8478723

[4] Horbach SER, Lokhorst MM, Saeed P, de Goüyon Matignon Pontourau CMF, Rothová A, van der Horst CMAM. Sclerotherapy for low-flow vascular malformations of the head and neck: a systematic review of sclerosing agents. J Plast Reconstr Aesthet Surg. 2016;69:295–304. 26723834 10.1016/j.bjps.2015.10.045

[5] Bolzan AD, Bianchi MS. DNA and chromosome damage induced by bleomycin in mammalian cells: an update. Mutat Res Rev Mutat Res. 2018;775:51–62. 29555029 10.1016/j.mrrev.2018.02.003

[6] Schmidt VF, Cangir O, Meyer L, et al. Outcome of bleomycin electrosclerotherapy of slow-flow malformations in adults and children. Eur Radiol. 2024;34:6425–34. 38627287 10.1007/s00330-024-10723-6 PMC11399160

[7] Horbach SER, van de Ven JS, Nieuwkerk PT, Spuls PI, van der Horst C, Reekers JA. Patient-reported outcomes of bleomycin sclerotherapy for low-flow vascular malformations and predictors of improvement. Cardiovasc Intervent Radiol. 2018;41:1494–504. 29948003 10.1007/s00270-018-1999-8 PMC6132854

[8] Kumar P, Nagarajan A, Uchil PD. Electroporation. Cold Spring Harb Protoc. 2019. 10.1101/pdb.top096271. 31575795 10.1101/pdb.prot095455

[9] Mir LM, Gehl J, Sersa G, et al. Standard operating procedures of the electrochemotherapy: instructions for the use of bleomycin or cisplatin administered either systemically or locally and electric pulses delivered by the CliniporatorTM by means of invasive or non-invasive electrodes. Eur J Cancer Suppl. 2006;4:14–25.

[10] Probst U, Fuhrmann I, Beyer L, Wiggermann P. Electrochemotherapy as a new modality in interventional oncology: a review. Technol Cancer Res Treat. 2018;17:1533033818785329. 29986632 10.1177/1533033818785329 PMC6048674

[11] Tasu JP, Tougeron D, Rols MP. Irreversible electroporation and electrochemotherapy in oncology: state of the art. Diagn Interv Imaging. 2022;103:499–509. 36266192 10.1016/j.diii.2022.09.009

[12] O’Donoghue N, Mowatt D, Sykes AJ. Electrochemotherapy and ablative therapies in non-melanoma skin cancer. Clin Oncol (R Coll Radiol). 2019;31:e1–9. 31543301 10.1016/j.clon.2019.08.010

[13] Muir T, Wohlgemuth WA, Cemazar M, et al. Current operating procedure (COP) for bleomycin electrosclerotherapy (BEST) of low-flow vascular malformations. Radiol Oncol. 2024;58:469–79. 39608012 10.2478/raon-2024-0061 PMC11604259

[14] Filippiadis DK, Binkert C, Pellerin O, Hoffmann RT, Krajina A, Pereira PL. Cirse quality assurance document and standards for classification of complications: the Cirse classification system. Cardiovasc Intervent Radiol. 2017;40:1141–6. 28584945 10.1007/s00270-017-1703-4

[15] Schmidt VF, Cangir O, Meyer L, et al. Outcome of bleomycin electrosclerotherapy of slow-flow malformations in adults and children. Eur Radiol. 2024. 10.1007/s00330-024-10723-6. 38627287 10.1007/s00330-024-10723-6 PMC11399160

[16] Wohlgemuth WA, Müller-Wille R, Meyer L, et al. Bleomycin electrosclerotherapy in therapy-resistant venous malformations of the body. J Vasc Surg Venous Lymphat Disord. 2021;9:731–9. 33045393 10.1016/j.jvsv.2020.09.009

[17] Kostusiak M, Murugan S, Muir T. Bleomycin electrosclerotherapy treatment in the management of vascular malformations. Dermatol Surg. 2022;48:67–71. 34608081 10.1097/DSS.0000000000003220

[18] Bini A, Topalidis C, Koletsa T, Papas A, Demiri E, Pavlidis L. The role of bleomycin sclerotherapy in venous malformation management: a narrative review. Life Basel. 2025. 10.3390/life15101553. 41157225 10.3390/life15101553 PMC12565142

[19] Hay J, Shahzeidi S, Laurent G. Mechanisms of bleomycin-induced lung damage. Arch Toxicol. 1991;65:81–94. 1711838 10.1007/BF02034932

[20] Della Latta V, Cecchettini A, Del Ry S, Morales MA. Bleomycin in the setting of lung fibrosis induction: from biological mechanisms to counteractions. Pharmacol Res. 2015;97:122–30. 25959210 10.1016/j.phrs.2015.04.012

[21] Muir T, Bertino G, Groselj A, et al. Bleomycin electrosclerotherapy (BEST) for the treatment of vascular malformations. An International Network for Sharing Practices on Electrochemotherapy (InspECT) study group report. Radiol Oncol. 2023;57:141–9. 37341196 10.2478/raon-2023-0029 PMC10286891

