# Supplementary material for: Prospective analysis of bleomycin electrosclerotherapy for clinical outcome and volume reduction in therapy refractory slow-flow malformations
Source: CVIR Endovasc. 2025 Dec 31;8:121. doi: 10.1186/s42155-025-00641-z (PMC12756204; doi:10.1186/s42155-025-00641-z)
Supplement: Supplementary file 1 — Supplementary Material 1. [file 42155_2025_641_MOESM1_ESM.docx]

**Supplementary File 1**

**Study Design and Patient Characteristics**

The study was approved by the local Institutional Review Board (approval no. BLINDED) and conducted in compliance with the Declaration of Helsinki. Informed consent, relative to participation in the procedure and the study, was obtained from all patients. Diagnoses were established based on patient history, MRI, ultrasound and clinical examination. Symptom evaluation was standardized, and all patients were examined using the same protocol. Patients were questioned and clinical records were reviewed, covering disease history, pain scale assessments as well as symptoms such as swelling, skin discolorations, restriction of motion, sensory impairment, thrombosis, and hypotrophy. In total 35 lesions were treated, of which 27 (77.1%) were venous malformations and 8 (22.9%) were veno-lymphatic malformations.
