# Supplementary material for: Prospective analysis of bleomycin electrosclerotherapy for clinical outcome and volume reduction in therapy refractory slow-flow malformations
Source: CVIR Endovasc. 2025 Dec 31;8:121. doi: 10.1186/s42155-025-00641-z (PMC12756204; doi:10.1186/s42155-025-00641-z)
Supplement: Supplementary file 2 — Supplementary Material 2. [file 42155_2025_641_MOESM2_ESM.docx]

**Supplementary File 2**

**Bleomycin Electrosclerotherapy**

The indication for BEST was determined by interdisciplinary consensus at the tertiary care center of BLINDED. Needles with a specific electrode design (linear, hexagonal, finger, and freely positionable) were selected based on the lesion's location (superficial or deep) and on the size. For example, the hexagonal electrode was chosen for patients with larger and deeper lesions, while the finger electrode was preferred for smaller and superficial lesions. Electrodes were positioned side by side within the margins of the vascular malformation to ensure complete coverage of the lesion with repetitive punctures while avoiding relevant gaps. To apply the electric pulses necessary for reversible electroporation, the electrodes were connected to the generator outputs of the electroporation system (Cliniporator VITAE; IGEA SpA, Carpi, Italy) (Figure 2).
