# Supplementary material for: Prospective analysis of bleomycin electrosclerotherapy for clinical outcome and volume reduction in therapy refractory slow-flow malformations
Source: CVIR Endovasc. 2025 Dec 31;8:121. doi: 10.1186/s42155-025-00641-z (PMC12756204; doi:10.1186/s42155-025-00641-z)
Supplement: Supplementary file 3 — Supplementary Material 3. [file 42155_2025_641_MOESM3_ESM.docx]

**Supplementary File 3**

**MRI Volumetric Analysis**

All patients underwent MRI on a 3 Tesla whole-body MR scanner (Magnetom Skyra, Siemens Healthineers, Erlangen, Germany) using the following standardized sequence protocol:

- Axial and coronal fat-saturated T2-weighted STIR (short tau inversion recovery)
- Axial T2-weighted TSE (turbo spin-echo)
- Non-contrast axial T1-weighted TSE
- Time-resolved contrast-enhanced TWIST (Time-resolved angiography With Interleaved Stochastic Trajectories) MR angiography
- Post-contrast fat-saturated gradient-echo T1-weighted high-resolution 3D VIBE (volumetric interpolated breath-hold examination)

To ensure consistency and reproducibility, all measurements were performed on T2-weighted images, which provide optimal delineation of slow-flow vascular malformations. In particular, the lesion volume was assessed using axial and coronal T2w STIR sequences, where slow-flow malformations typically appear as areas of marked hyperintensity. The lesions were manually delineated at baseline before the first BEST intervention and again at the post-therapy follow-up visit. Given the irregular morphology of VMs, the lesion volume was approximated using a rotational ellipsoid formula ($V =\frac{\pi}{6}\times d_{e}^{2}\times d_{p}$), in which $d_{e}$ represents the equatorial diameter as well as $d_{p}$ represents the polar diameter. We subsequently calculated the percentage change in volume between the follow-up MRI scans and the baseline scans obtained before the intervention. MRI scans, combined with ultrasound examination, were performed at each follow-up visit, and lesion volumes were quantified at each time point relative to the baseline volume, established prior to the initiation of BEST treatment.
